# Supplementary material for: Patterns of Intron Gain and Loss in Fungi
Source: PLoS Biol. 2004 Nov 30;2(12):e422. doi: 10.1371/journal.pbio.0020422 (PMC532390; doi:10.1371/journal.pbio.0020422)
Supplement: Table S1 — Also available at http://genes.mit.edu/NielsenEtAl/. (4.3 MB ZIP). [file pbio.0020422.st001.zip › NielsenEtAl/html/140.html]

AN6346.1.NCU04579.1.MG05345.1.FG02056.1


```
 CLUSTAL W (1.82) Multiple Sequence Alignments - Introns Inserted


Sequence 1: MG05345.1	594 aa
Sequence 2: FG02056.1	598 aa
Sequence 3: NCU04579.1	596 aa
Sequence 4: AN6346.1	613 aa
Alignment Length: 616 aa
Number Identitical Residues: 390 aa
Alignment Score (without introns) 16932


MG05345.1 	-MSSNLLRARVPKAL--AATRSHA2ALFSTTSRR--------AEQLNKTSAKITQPKSQG
NCU04579.1	MLAPSLLRAQAPRAL--ASARLSL2RSLSTTPRRYNA----QEKQLNKVSANITQPKSQG
FG02056.1 	MLSRSLLRSRAVGAFP-LSARNHG2RFLSTTSIRS-------DDKLNKISSNITQPKAQG
AN6346.1  	-MLLSQTRGRLPSALRSLANRAAI2RPISTTLPRQKASPKDDEPVLNKVSRHITQPVSQG
          	 :  .  *.:   *: : : *      :***  * .::....   *** * :**** :**

MG05345.1 	ASQAMLYATGLTEEDMNKPQVGISSVWYEGNPCNMHILKLSERIRDSVKAANLVPMRFNT
NCU04579.1	ASQAMLYATGLNEDDMNKAQVGISSVWYEGNPCNMHLLDLSGLVKESVAKAGLVPMRFNT
FG02056.1 	ASQAMLYATGLSEADMNKAQVGISSVWYEGNPCNMHLMDLSAHVKESVAKAGLIPYRFNT
AN6346.1  	ASQAMLYATGLTEADMNKAQVGISSVWYNGNPCNMHLLDLNNRVREGVQKAGLIGYQFNT
          	***********.* ****.*********:*******::.*.  :::.*  *.*:  :***

MG05345.1 	IGVSDGISMGTTGMRYSLQSREIIADSIETVMQGQWYDANISIPGCDKNMPGVLIALGRV
NCU04579.1	IGVSDGISMGTTGMRYSLQSREIIADSIETVMNGQWYDANISLPGCDKNMPGVLIAMGRV
FG02056.1 	IGVSDGISMGTTGMRYSLQSREIIADSVETVMNGQWYDANVSLPGCDKNMPGVAIAMGRV
AN6346.1  	IGVSDGISMGTSGMRYSLQSRDLIADSIETVMGGQWYDANISIPGCDKNMPGVLMAMGRV
          	***********:*********::****:**** *******:*:********** :*:***

MG05345.1 	NRPSLIVYGGTIKPGCS-QKG-EPIDIVSAFQAYGQYLTGEITEEQRFDIIRNACPGGGA
NCU04579.1	NRPSIMVYGGTIKPGCN-MKG-ENIDIVSAFQAYGQYISGEIDEKQRFDIIRNACPGGGA
FG02056.1 	NRPSIMVYGGTIKPGCT-KQG-ESIDIVSAFQAYGQYITGEITEEQRFDIIRNACPGGGA
AN6346.1  	NRPSLMVYGGTIKPGCASMQGNADIDIVSAFQAYGQFISGEINEPQRFDIIRHACPGGGA
          	****::********** : :*.  ************:::*** * *******:*******

MG05345.1 	CGGMYTANTLASAIETLGMSLPGSSSNPAEHPSKLAECDQVGEAIKNILREDVRPRDIMT
NCU04579.1	CGGMYTANTMATAIETLGMTLPGSSSYPAESPEKKNECLSVGEAIKNLLREDIRPTDILT
FG02056.1 	CGGMYTANTMATAIETLGLTLPGSSSSPAEDPSKIAECEAVGPAIRNILKEDIRPRDIMT
AN6346.1  	CGGMYTANTMATAIEVMGMTLTGSSSNPAESQAKYDECLRAGEAIKRLLVEDIRPSDIMT
          	*********:*:***.:*::*.**** ***   *  **  .* **:.:* **:** **:*

MG05345.1 	RQAFENAMVVVSILGGSTNAVLHLLAVADAVGVKLTIDDFQAVSDRTPLLADLKPSGKYV
NCU04579.1	RQAFENAMIVVNILGGSTNAVLHLIAIADSVGIKLTIDDFQAVSDRTPFLADLKPSGKWV
FG02056.1 	RQAFENAMIVTTILGGSTNAVLHLIAIADSVGIKLDIEDFQKVSDRTPFLADLKPSGKWV
AN6346.1  	RQAFENAMVVVNITGGSTNAVLHLIAIADSVGIKLTIDDFQAVSDRTPFLADLKPSGKYV
          	********:*..* **********:*:**:**:** *:*** ******:*********:*

MG05345.1 	MEDVHKIGGTPSLLRFLAKEGLIDASGITVTGKTMKENLDKYPDFPADQPIIRPLSNPLK
NCU04579.1	MEDLSKIGGTPALLKFLLKEGILDGSGITSTGKTMKENVEKFPDFPTDQAIIRPLSNPIK
FG02056.1 	MADMHKIGGTPALLKFLLKEGIIDGSGITVTGKTMKQNVEELPGFPEDQTIIRPLSNPIK
AN6346.1  	MADLHNIGGTPSLLKFLLKEGVIDGSGITVTGETLAKNLEKVPDFPEDQKIIRPFSNPIK
          	* *: :*****:**:** ***::*.**** **:*: :*::: *.** ** ****:***:*

MG05345.1 	STGHIQILRGSLAPGGSVGKITGKEGLQFTGKARCYDCEDDFIESLERGEIKKGEKTVVI
NCU04579.1	ETGHIQILRGSLAPGGSVGKITGKEGLRFEGKARCFDYEDGFIEALERGEIKKGEKTVVV
FG02056.1 	PTGHIQILRGSLAPGGCVGKITGKEGLRFEGKARVYDSEPAFISSLEAGEIKKGEKTVVI
AN6346.1  	ETGHIQILRGSLAPGGCVGKITGKEGTVFTGKARVFNHEDDFIAALERKEITKDEQTVVV
          	 ***************.*********  * **** :: *  ** :**  **.*.*:***:

MG05345.1 	IRYEGPKGGPGMPE~MLKPSSAIMGAGLGKDVALITDGRFSGGSHGFLIGHVVPEAMEGG
NCU04579.1	IRYEGPKGGPGMPE~MLKPSSAIMGYGLGKDVALITDGRFSGGSHGFLIGHIVPEAMEGG
FG02056.1 	IRYDGPKGGPGMPE~MLKPSSAIMGAGLGQDVALLTDGRFSGGSHGFIIGHIVPEAMEGG
AN6346.1  	IRYTGPKGGPGMPE1MLKPSSALMGAGLGQTCALITDGRFSGGSHGFLIGHIVPEAAVGG
          	*** ********** *******:** ***:  **:************:***:****  **

MG05345.1 	PIALVRDGDTITIDAEKRVIDTDVSDKTMAERRAEWKAPPIR--------ETRGTLAKYA
NCU04579.1	PIGLVRDGDTIVIDAEKKVLDLEVPEEELAKRRKEWKAPEPK--------AKRGTLRKYA
FG02056.1 	PIALVEDGDTIVIDAESRAIDLVVPEAEVDRRRKAWKAPAPR--------YTKGTLSKYA
AN6346.1  	PIGLVHDGDVITIDAEKRVLDLDVDEAELAKRRKQWEADKAAGKLPQTGLNLRGTLGKYA
          	**.**.***.*.****.:.:*  * :  : .**  *:*    .. ..:.   :*** ***

MG05345.1 	ALVSDASSGCVTDKVAR-
NCU04579.1	QLVKDASSGCVTDA----
FG02056.1 	RLVTNASEGCVTDSGLKN
AN6346.1  	RNVKDASSGCITDAFD--
          	  *.:**.**:**
```
